# Serum free fatty acid elevation is related to acute kidney injury in primary nephrotic syndrome

**DOI:** 10.1080/0886022X.2022.2105232

**Published:** 2022-08-01

**Authors:** Lili Zhang, Li Cui, Chunmei Li, Xiangzhong Zhao, Xiaoying Lai, Jing Li, Teng Lv

**Affiliations:** a Department of Nutrition, The Affiliated Hospital of Qingdao University, Qingdao, China; b Department of Nephrology, The Affiliated Hospital of Qingdao University, Qingdao, China; c Medical Research Center, The Affiliated Hospital of Qingdao University, Qingdao, China; d Department of Gynecology, The Affiliated Hospital of Qingdao University, Qingdao, China

**Keywords:** Acute kidney injury, fatty acid, nephrotic syndrome, biomarker, acute tubular necrosis

## Abstract

The aim of this research was to examine the clinical characteristics of acute kidney injury (AKI) in primary nephrotic syndrome (NS) and discuss the relationship between serum lipids and AKI. A total of 1028 patients diagnosed with primary NS with renal biopsy results were enrolled in this study. The patients were divided into AKI (*n* = 81) and non-AKI (*n* = 947) groups, and their characteristics were compared using a propensity score analysis for the best matching. Serum free fatty acid (FFA) was an independent predictor for AKI in the postmatch samples (*p* = 0.011). No significant difference in FFA levels was observed among AKI stages or different pathological types in the AKI and non-AKI groups. The AUC (area under the ROC curve) was 0.63 for FFA levels to distinguish AKI. In primary NS, elevated FFA levels tend to be related to a high risk of AKI. FFAs have diagnostic value and may serve as biomarkers for AKI in NS.

## Introduction

Nephrotic syndrome (NS) is characterized by hypoalbuminemia and massive proteinuria, and acute renal injury (AKI) is one of the severest complications. Due to the aggressiveness of the disease, renal interstitial edema or other surrounding factors of pathophysiology and treatment [1,2], patients with NS are prone to develop AKI. As part of the initial clinical manifestations, AKI has been reported to occur in 23%–47% of minimal change diseases [3–6], whereas a lower incidence has been reported in membranous nephropathy (MN) [7]. In the majority of cases, AKI accompanied by primary NS is reversible. For most patients, renal function can recover completely or partially in 4–8 weeks [8]. In some cases, however, renal function is irreversibly damaged [9–14]. Treatment of AKI may require dialysis, especially when it is complicated by severe electrolyte imbalance, positive fluid balance or overt uremic symptoms until oliguria resolution. AKI is widely known to be linked to high mortality and morbidity, resulting in an increased risk of subsequent chronic kidney disease [15,16]. Lipid metabolism disorder is one of the most common manifestations of NS. As a product of fat catabolism, elevated free fatty acids are thought to be associated with a rapid decline in renal function and may predict renal function decline in the early stages of chronic kidney disease [1]. To date, there is no relevant report on the clinical study of FFA in nephrotic syndrome. Here, we discuss the clinical and pathological characteristics of AKI and attempt to clarify the related risk factors and tendencies of AKI in NS to better understand the disease and its relationship with FFA.

## Materials and methods

### Patients

**Figure 1.**
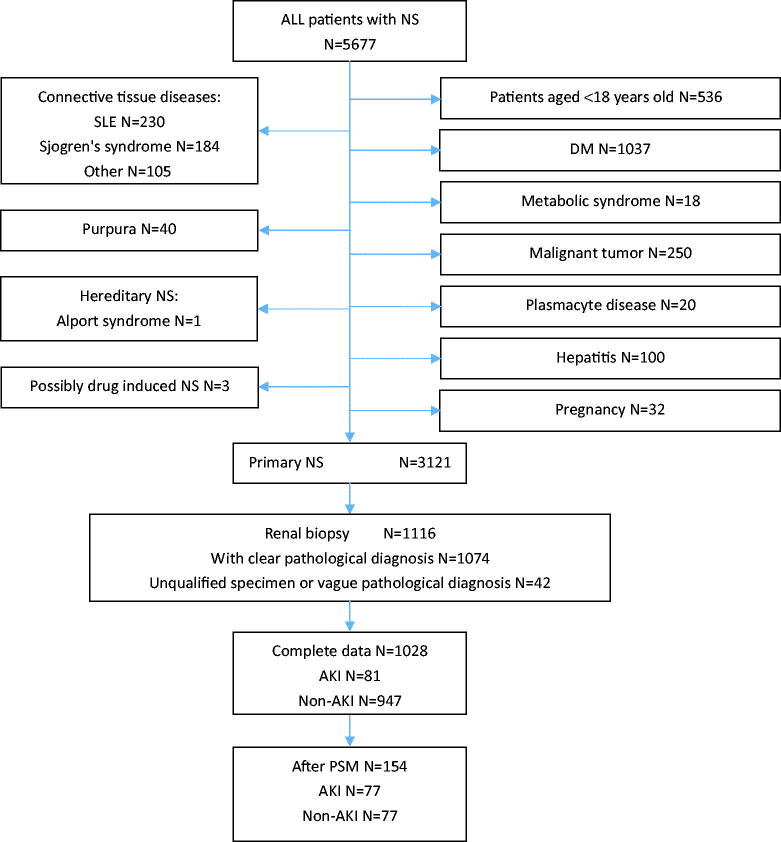
Flow diagram of the patient selection. PSM, propensity score matching.

This was a retrospective, single-center, and observational cohort study. This study was approved by the ethics committee of the Affiliated Hospital of Qingdao University (approval number: QYFY WZLL 26502). Figure 1 shows a flow diagram of the patient selection process. From 2012.12 to 2022.4, data from hospitalized patients older than 18 years were extracted from the Affiliated Hospital of Qingdao University electronic medical record system. During the observation period, the four hospital districts of the Affiliated Hospital of Qingdao University had a total of 3700 beds, of which 93 were in the nephrology department. A total of 5677 patients were diagnosed with NS. Patients with diseases that can lead to secondary NS, such as metabolic syndrome, hepatitis, diabetes, tumors, Henoch-Schonlein purpura, connective tissue disease, and other immune system diseases, were excluded. Those with hereditary NS were excluded. We collected 3121 cases involving primary NS diagnosis. Among them, 1074 patients underwent renal biopsy with definitive pathological diagnosis. Those with incomplete data were excluded. Ultimately, we collected 1028 patients diagnosed with primary NS. Information collected included the following: age; sex; diagnosis; BMI; systolic blood pressure (SBP); pathological type; and laboratory serum testing, including serum albumin, 24-h urine proteinuria, triglycerides (TGs), total cholesterol (TCHO), low-density lipoprotein cholesterol (LDL), high-density lipoprotein cholesterol (HDL), free fatty acids (FFAs), urea and creatinine (SCr). The diagnostic criterion of AKI for this study was an increase in serum creatinine to 1.5 times the baseline value within 7 days [17,18]. AKI was classified into 3 stages according to the 2012 KDIGO definition [19]. We did not cite the urine output criteria because hourly urine volume was difficult to obtain from medical records. This study was approved by the Medical Ethics Committee of the Affiliated Hospital of Qingdao University.

### Laboratory testing

Bromocresol green spectrophotometry was used to determine serum albumin. Serum TG was determined using the GPO-PAP method and serum TCHO by the CHOD-POD method. HDL was assessed by the selective inhibitory direct method, and LDL was evaluated by the direct method. Serum FFA (nonesterified fatty acids) was examined by the enzyme endpoint method. Serum urea was detected by the urease glutamate dehydrogenase method and serum creatinine by the picric acid method. The pyrogallol red method was used to determine the concentration of urinary protein. A Beckman Coulter Au5400 and Au5800 (Beckman Coulter, Inc. S. Kraemer Boulevard Brea, CA 92821, USA) were used in this study.

### Statistical analysis

Continuous variable data were evaluated by the one-sample Kolmogorov–Smirnov test. The results are presented as median values (25th percentile, 75th percentile) for continuous variables with a nonnormal distribution. Categorical variables are presented as percentages. We used propensity score matching (PSM) to match AKI patients and non-AKI patients to minimize the impact of other confounding factors on the results. AKI patients were matched with non-AKI patients at a ratio of 1:1 based on sex, age, infection, proteinuria, SBP, FFA, BMI, TCHO, TG, LDL, HDL and albumin. The PSM matching tolerance was set as 0.02. The matching success ratio was 95%. Before and after matching, differences in continuous variables with nonnormal distributions were compared by the Mann–Whitney U test. The chi-square test or Fisher's exact test was used to compare the distributions of categorical variables. Baseline variables in univariate analysis showing a significant correlation between AKI and non-AKI were entered into the multivariate model (*p* ≤ 0.05). Multivariate logistic regression analysis was used to evaluate the odds ratios (ORs) and 95% confidence intervals (CIs) between AKI and non-AKI patients. The diagnostic ability of serum FFA levels was further evaluated by receiver operating characteristic (ROC) curve analysis. SPSS software 26.0 and GraphPad Prism 7.0 were applied to perform all statistical analyses. *p* < 0.05 was regarded as a statistically significant difference.

## Results

A total of 1028 patients aged ≥18 years with primary NS were enrolled. Overall, 81 patients (7.9%) presented with AKI. All of them underwent renal biopsy and had a definite pathological diagnosis. The basic features of the patients in the prematch and postmatch models are shown in Table 1. After PSM analysis, 77 patients were matched. The prematch analysis showed that AKI patients were older than those in the non-AKI group (60 years vs. 52 years, *p* = 0.011), with a higher percentage of males (78% vs. 63%, *p* = 0.009), a greater rate of infection (32% vs. 7%, *p* < 0.001), higher SBP (147 mmHg vs. 135, *p* < 0.001), and greater BMI (27.2 vs. 25.5 kg/m^2^, *p* = 0.003). The test results showed greater 24-h urine proteinuria (6.1 g vs. 4.4 g, *p* < 0.001) and elevated FFA levels (0.39 mmol/L vs. 0.33 mmol/L, *p* < 0.001) in the AKI group compared to the non-AKI group, whereas albumin (21.2 vs.23.6 mmol/L, *p* < 0.001) and HDL levels (1.57 mmol/L vs. 1.71 mmol/L, *p* = 0.006) were obviously decreased. After controlling for sex, age, infection, proteinuria, SBP, FFA, BMI, TCHO, TG, LDL, HDL and albumin and test results using the PSM approach, the AKI group still showed elevated FFA levels (0.39 mmol/L vs. 0.31 mmol/L, *p* ＝ 0.006).

**Table 1.** Baseline clinical characteristics before and after propensity score matching.

Variables	Before	Matching	*p*	After	Matching	*p*
	Non-AKI (*n* = 947)	AKI (*n* = 81)		Non-AKI (*n* = 77)	AKI (*n* = 77)	
Age (years)	52 (37–63)	60 (40–72)	0.011	57 (42–64)	60 (40–71)	0.346
Male	600 (63%)	63 (78%)	0.009	63 (82%)	60 (78%)	0.547
Infection	69 (7%)	26 (32%)	<0.001	22 (29%)	25 (32%)	0.600
Proteinuria (g)	4.4 (2.5–6.7)	6.1 (4.0–10.1)	<0.001	6.2 (4.0–9.7)	5.7 (4.0–9.3)	0.803
SBP (mmHg)	135 (124–150)	147 (133–164)	<0.001	150 (135–168)	147 (132–164)	0.590
BMI (kg/m2)	25.5 (23.1–28.0)	27.2 (23.8–29.8)	0.003	26.4 (24.4–28.6)	27.1 (23.6–29.8)	0.490
FFA (mmol/L)	0.33 (0.22–0.46)	0.39 (0.30–0.59)	<0.001	0.31 (0.23–0.49)	0.39 (0.30–0.59)	0.006
Urea (mmol/L)	5.67 (4.58–7.16)	14.3 (9.5–19.3)	<0.001	6.28 (5.25–9.55)	14.65 (9.56–19.87)	<0.001
SCr (µmol/L)	70 (57–85)	188 (147–270)	<0.001	79 (65–96)	190.3 (152.0–274.0)	<0.001
TCHO (mmol/L)	7.54 (6.07–9.54)	7.85 (5.56–9.87)	0.980	7.48 (5.57–9.08)	7.78 (5.43–9.80)	0.609
TG (mmol/L)	2.01 (1.41–2.87)	2.25 (1.49–3.10)	0.304	1.97 (1.29–2.65)	2.22 (1.46–3.04)	0.394
LDL (mmol/L)	4.80 (3.55–6.42)	4.93 (3.34–6.75)	0.823	4.65 (3.38–6.27)	4.85 (3.34–6.75)	0.632
HDL (mmol/L)	1.71 (1.37–2.18)	1.57 (1.27–1.83)	0.006	1.66 (1.22–2.01)	1.57 (1.26–1.80)	0.254
Albumin (g/L)	23.6 (19.0–28.8)	21.2 (16.1–25.5)	<0.001	21.6 (18.1–25.6)	21.3 (16.2–25.9)	0.264

Table 2 shows that before PSM analysis, male sex, higher infection, older age, larger proteinuria, higher systolic pressure, higher FFA, and lower albumin were associated with AKI according to univariate analysis. Multivariate logistic regression analyses revealed a higher rate of infection, higher systolic pressure, elevated serum FFA, and greater 24-h proteinuria in association with a tendency toward AKI. After PSM analysis (Table 3), univariate analysis showed a correlation of higher FFA with AKI. A FFA value of 1 mmol/L resulted in an 82.18% risk of AKI [OR (95% CI): 9.218 (1.653–51.419), *p* = 0.011]; that is, FFA values increased by 0.1 mmol/L, and AKI incidence increased by 8.218%.

**Table 2.** Univariate and multivariate logistic regression analyses of risk factors for AKI in NS patients in prematched samples.

	Univariate analysis		Multivariate analysis	
	OR (95%CI)	*p*	OR (95%CI)	*p*
Sex	0.494 (0.288–0.848)	0.011	0.648 (0.365–1.151)	0.139
Infection	0.166 (0.098–0.282)	<0.001	0.206 (0.116–0.364)	<0.001
24 h proteinuria (g/L)	1.120 (1.070–1.172)	<0.001	1.093 (1.034–1.155)	0.002
Age (years)	1.019 (1.004–1.034)	0.012	1.012 (0.996–1.027)	0.135
Systolic pressure (mmHg)	1.024 (1.013–1.034)	<0.001	1.021 (1.010–1.033)	<0.001
BMI (kg/m^2^)	1.005 (0.991–1.019)	0.483		
FFA (mmol/L)	3.970 (1.695–9.298)	0.002	3.343 (1.227–9.105)	0.018
TCHO (mmol/L)	0.989 (0.915–1.070)	0.790		
TG (mmol/L)	1.016 (0.904–1.142)	0.789		
LDL (mmol/L)	0.978 (0.885–1.081)	0.660		
HDL (mmol/L)	1.128 (0.883–1.440)	0.335		
Albumin (g/L)	0.937 (0.902–0.973)	0.001	0.955 (0.913–1.000)	0.050

**Table 3.** Univariate logistic regression analyses of risk factors for AKI in NS patients in postmatch samples.

	Univariate analysis	
	OR (95%CI)	*p*
Sex	0.784 (0.356–1.730)	0.547
Infection	0.832 (0.419–1.654)	0.600
24 h proteinuria (g/L)	1.009 (0.940–1.083)	0.805
Age (years)	1.007 (0.988–1.026)	0.480
Systolic pressure (mmHg)	0.996 (0.983–1.008)	0.498
BMI (kg/m^2^)	1.038 (0.946–1.140)	0.428
FFA (mmol/L)	9.218 (1.653–51.419)	0.011
TCHO (mmol/L)	1.024 (0.906–1.158)	0.705
TG (mmol/L)	1.091 (0.873–1.364)	0.442
LDL (mmol/L)	1.014 (0.868–1.185)	0.857
HDL (mmol/L)	0.950 (0.645–1.400)	0.795
Albumin (g/L)	0.978 (0.933–1.025)	0.353

Table 4 shows that 46% of AKI the cases were at stage 1, 30% at stage 2, and 25% at stage 3. The FFA level of stage 1 was 0.38 (0.31–0.50), that of stage 2 was 0.46 (0.26–0.62), and that of stage 3 was 0.37 (0.23–0.63). There was no significant difference in FFA level among the AKI stages. The AKI incidence was 12.5% (19/152) in MCD, 3.27% (21/643) in MN, 22.95% (14/61) in FSGS, 15.70% (19/121) in IgAN, 11.11% (2/18) in MCGN and 18.18% (6/33) in MesPGN. AKI incidence was highest in FSGS patients and lowest in MN patients. As shown in Table 5, in the AKI group, MCD accounted for 23.5% (19/81), MN for 25.9% (21/81), FSGS for 17.3% (14/81), IgAN for 23.5% (19/81), MesPGN for 7.4% (6/81), and MCGN for 2.5% (2/81) of cases. In the non-AKI group, MCD accounted for 14% (133/947), MN for 65.7% (622/947), FSGS for 5.0% (47/947), IgAN for 10.8% (102/947), MesPGN for 2.9% (27/947), and MCGN for 1.7% (16/947) of cases. The chi-square test and Fisher's exact test indicated that the AKI group had a higher proportion of MCD, FSGS, IgAN and MesPGN and a lower proportion of MN than the non-AKI group (*p* < 0.05). These results suggest that among various pathological types, patients with MN are least prone to AKI development. Table 5 also shows that there were no statistically significant differences in FFA level among the various pathological types of AKI (*p* = 0.510) and non-AKI (*p* = 0.532), suggesting that serum FFA levels were not related to pathological type in this study.

**Table 4.** Comparison of FFA levels among AKI stages (*n* = 81).

	*n*	FFA	*p*
Stage 1	37 (46%)	0.38 (0.31–0.50)	0.777
Stage 2	24 (30%)	0.46 (0.26–0.62)	
Stage 3	20 (25%)	0.37 (0.23–0.63)	

**Table 5.** Comparison of FFA levels among different pathological types in the AKI group and non-AKI group.

Pathological type	AKI (*n*) *N* = 81	Non-AKI (n) *N* = 947	p*	AKI-FFA (mmol/L)	p**	Non-AKI FFA (mmol/L)	p***
MCD	19 (23.5%)	133 (14%)	0.022	0.49 (0.27–0.63)	0.510	0.32 (0.22–0.46)	0.532
MN	21 (25.9%)	622 (65.7%)	<0.001	0.38 (0.29–0.48)		0.33 (0.22–0.45)	
FSGS	14 (17.3%)	47 (5.0%)	<0.001	0.50 (0.32–0.65)		0.34 (0.22–0.52)	
IgAN	19 (23.5%)	102 (10.8%)	0.001	0.39 (0.31–0.55)		0.33 (0.20–0.49)	
MesPGN	6 (7.4%)	27 (2.9%)	0.026	0.35 (0.17–0.47)		0.35 (0.25–0.53)	
MCGN	2 (2.5%)	16 (1.7%)	0.647	0.18–0.48		0.22 (0.17–0.33)	

*p comparison of pathological results between AKI and non-AKI groups; **p comparison of FFA levels among different pathological types in the AKI group; ***p comparison of FFA levels among different pathological types in the non-AKI group.

**Figure 2.**
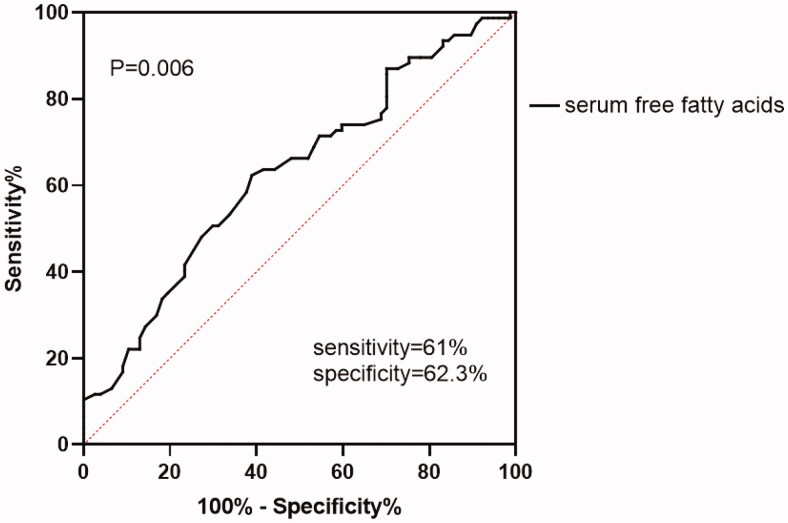
ROC curves of FFA levels between the AKI group and non-AKI group (AUC = 0.63).

As shown in Figure 2, the area under the ROC curve was 0.63 (95% CI, 0.54–0.71) in the AKI group, with 61% sensitivity and 62.3% specificity, indicating that serum FFA has a weak diagnostic value got discriminating AKI patients from non-AKI patients.

## Discussion

The occurrence of AKI in adults with NS was 7.9% in our survey. According to previous studies, a number of risk factors, such as older age, hypertension, male sex, severe proteinuria, severely decreased serum albumin, and atherosclerosis, were found to lead to AKI in NS [20–23]. In addition, nephrotoxic medication exposure correlates with AKI [24]. In our study, multivariate logistic regression analyses clarified that infection, higher systolic blood pressure, higher levels of FFAs, and proteinuria were associated with an increased risk of AKI. In postmatch sample analysis, FFA still showed statistical significance, which indicated that FFA may serve as an indicator for the detection of AKI.

FFAs are produced by hydrolysis of triglycerides. As metabolites, fatty acids play a powerful intermediary role in many biological processes. As a key component of phospholipids and glycolipids, fatty acids are important fuel molecules in cell structure and function. Under fasting conditions, fatty acids can also provide energy for cells [25–27]. Abnormal fatty acid metabolism may lead to abnormal pathological metabolic conditions, such as hyperthyroidism, severe liver dysfunction, obesity, insulin resistance and type 2 diabetes mellitus [28–33]. Such abnormalities of serum lipids and lipoproteins in NS are due to impaired clearance and altered biosynthesis [34]. In response to hypoproteinemia, the liver increases not only albumin formation but also lipid production [35,36]; thus, elevated serum lipid levels, including FFA levels, are closely related to decreased albumin levels in patients with NS. However, patients and animals with advanced CKD without nephrosis-range proteinuria also exhibit hypertriglyceridemia and elevated FFA levels [34], which suggests that increased FFA may be one of the manifestations of renal insufficiency rather than the result of decreased albumin. Indeed, a lack of albumin, which can act as an acceptor and carrier of FFA, may diminish intravascular lipolysis and reduce plasma FFA concentrations [37,38]. Therefore, an increase in FFA level can be used as an independent predictor for AKI in NS patients.

Despite no relevant report on the clinical study of FFA in NS, studies have shown that FFA is associated with a rapid decline in renal function and may predict renal function decline in the early stages of chronic kidney disease [1]. Excessive accumulation of FFA causes renal injury through different mechanisms. Several mechanisms, such as the inflammatory response, oxidative stress reaction, activated renin-angiotensin system, impaired insulin signal transduction, effects of nitric oxide production and endothelial cell apoptosis [39–45], are thought to be related to endothelial dysfunction mediated by elevated FFAs. Endothelial dysfunction and vascular injury are considered to be involved in the occurrence of AKI [46–48]. FFA can also cause inflammation and injury of podocytes and the renal tubulointerstitium. FFA can cause podocyte apoptosis, downregulate expression of markers and increase that of the inflammatory factors IL-18 and IL-1β and also induce NF-κB activation. FFA promotes expression of IL-6 and IL-18 and cell death in proximal renal tubular cells [40–42]. *In vitro* experiments have proven an association between FFA and renal tubulointerstitial injury and endothelial cell apoptosis. Our study confirmed that elevated FFAs are associated with acute renal injury in patients with NS. An animal experiment also showed that FFAs can affect proteinuria by directly altering podocyte function and viability [49]. Therefore, we believe that FFA is also involved in the occurrence of AKI in NS patients. Our results showed that the ROC value of FFA as a diagnostic criterion was 0.63, indicating that serum FFA has low accuracy as a biomarker of AKI, though serum FFA might be an indicator for the detection of AKI.

AKI incidence was highest in FSGS and lowest in MN. Compared with the non-AKI group, the proportion of MCD, FSGS, IgAN and MesPGN in the AKI group was higher, but it was lower in MN, which indicates that patients with MN are least prone to developing AKI, consistent with previous studies [6,7].

There are several limitations to the present research. First, this was an observational and retrospective cohort study, and some cases were excluded due to missing data. Second, this was a single-center study with a limited number of samples, which may have caused bias.

## Conclusions

In summary, elevated serum FFA levels might be associated with the risk of AKI in NS and may therefore be a supplement to current AKI detection.

## Data Availability

All data generated or analyzed during this study are included in this published article and its Online Resources.
